# An Enquiry into the Source from Whence the Symptoms of the Scurvy, and of Putrid Fevers Arise; and into the Seat Which Those Affections Occupy in the Animal Œconomy; with a View of Ascertaining a More Just Idea of Putrid Diseases Than Has Generally Been Formed of Them

**Published:** 1782

**Authors:** 


VI. *An Enquiry into the Source from whence the Symptoms of the Scurvy, and of putrid Fevers arise; and into the Seat which those Affections occupy in the Animal Œconomy; with a View of ascertaining a more just Idea of putrid Diseases than has generally been formed of them. By* Francis Milman, M. D. F. R. S. *Fellow of the Royal College of Physicians, and lately one of Dr, Radcliffe's travelling Physicians.* 8vo. Dodsley, *London*, 1782. 231 pages, 3s.

IT has been very properly observed by a learned and respectable modern writer, that "the more we know of the human body, "the more reason we find to believe that the "seat of diseases is not to be sought for "in

"in the blood, to the fenfible qualities of "which they feem to have very little relation *." The volume before us is founded on this idea, which the author has purfued with confiderable ingenuity, and illuftrated by a variety of interefting obfervations collected from different writers.

In his preface to the work Dr. Milman remarks, that fome authors have attempted to point out the fuppofed nature of the vitiated ftate of the blood, which has been faid to be putrid in one difeafe, and acrimonious in another. The term *putrid* when applied to the difeafes of the animal œconomy, he confiders as extremely vague and equivocal in its fignification. Where—he afks—are the inftances recorded of putrid blood drawn from the living body? or where is the phyfician who would affert, that a putridity of the vital circulating fluid would be for a moment compatible with animal life? He is of opinion then that putrid difeafes have been faid to be conftituted by a caufe which cannot exift, and he undertakes to prove that the doctrines which have been

* See Medical Tranfact. vol. II. page 505.

founded founded upon this idea, have been no lefs pernicious in their tendency, than groundlefs in their principle; and have biaffed improperly both our attempts to prevent, and our endeavours to cure thofe complaints.

As the hiftories of the fcurvy and of putrid fevers furnifh particular and ftriking proofs of thefe general affertions, he confines his inquiries chiefly to thofe two fubjects. The parts of the work which relate to the fcurvy were read in July 1780, as the Gulfton lecture at the college of Phyficians. His firft view was merely to difcover the caufes of Captain Cooke's fuccefs in preferving his men from the fcurvy, and to account for the fymptoms of that difeafe: but in extending his inquiry, experiments on the actual ftate of the blood, both in the fcurvy and in putrid fevers, made by the moft able and unprejudiced phyficians, are adduced to fhew how extremely miftaken thofe perfons have been, who have referred the proximate caufe of the former to a putrefaction of the blood, gradually accumulated; and of the latter, to a fudden corruption of it.

In the introduction to the work Dr. Milman, as a proof of the great fatality of the fcurvy, remarks remarks that in the war before the laſt, more perſons were ſaid to have been deſtroyed by this diſeaſe alone, than to have periſhed by the wreck of ſtorms, and the united efforts of our combined enemies. Great however—ſays he,—as is the number of ſeamen which have been carried off by this diſeaſe in a ſhort channel cruize, amounting frequently in an inconſiderable fleet to many hnndreds, a late celebrated navigator, with a company of one hundred and eighteen men, actually performed a voyage of three years and eighteen days, through all the various climates from 50°. north to 71°. ſouth lat. with the loſs only of one man. He propoſes, therefore, however humiliating it may be to the medical profeſſion, to correct the errors of our ſyſtems, by the experience and wiſdom of Captain Cooke.---The introduction concludes with a ſhort account of ſome of the moſt ſtriking features of the ſcurvy.

The work itſelf is divided into twelve chapters. In the firſt chapter, Dr. Milman treats of the prediſpoſing cauſes of ſcurvy, which he refers to a ſtate of body impaired by preceding illneſs; to the want or exceſs of exercise; to cold and moisture, or to a gloomy, sorrowful state of mind.

The occasional or exciting causes are the subject of the succeeding chapter. These exciting causes, we are told, are principally three, viz. a diet of difficult digestion; food containing but little nourishment, or, certain passions of mind. Each of these causes as well as those spoken of in the preceding chapter are treated at some length, and illustrated by remarks from Lind, Rouppe, Kramer and other practical writers.

Dr. Milman next treats of the means of preventing the scurvy. These he observes must be as various as the circumstances which predispose to it, or the causes which excite it. To aim at any universal rule of opposing its invasion, he thinks, would be equally absurd and empirical. The bark, chalybeates, &c. he observes, are well calculated to guard the convalescent or to secure a relaxed constitution; but in the cold regions of Russia, such medicines would be poor substitutes for warm baths and flannel cloathing, and on the other hand, these, which are of so much benefit to the Russians, would serve only to increase the weakness, and to haften the approach of the diforder to the infirm valetudinarian. By an attention to cleanlinefs, by guarding againft fatigue, and by providing at all times a plenty of frefh water, Captain Cooke's feamen lived with impunity on their falt provifions. And in the very northern regions, in Greenland and in Lapland, continues our author, notwithftanding the predifpofing cold of thofe climates, a diet of eafy digeftion, without the aid of any vegetable fubftance whatever, has afforded equal fafety.

Dr. Milman thinks it has arifen entirely from a neglect of thefe diftinctions, that our endeavours to prevent the fcurvy have generally been fruftrated. Inftead of attending to the means of guarding againft indolence, fatigue, cold, &c. we have been led by delufive theories to reprefent other things of lefs moment as matters of much greater confequence; and a greater ftrefs has been laid on the ufe of the robs of lemons, &c. than on the meafures which have been mentioned. Sailors, he obferves, feem to have been taught to rely too much upon us, and too little upon themfelves, and to expect that fecurity from medicines, which is only to be derived from their own conduct and good management.

In the fourth chapter our author treats of the proximate cause of scurvy, and after quoting the observations of Monchy, Lind, Rouppe and others, he concludes that it does not consist in a putridity of the blood, as Sir John Pringle was led by his experiments to suppose.

Dr. Milman next takes occasion to speak of the irritability of the muscular fibres, and of the causes which lessen this power. He then proceeds to consider the symptoms of the scurvy as described by Boerhaave and Van Swieten, all of which he contends sufficiently prove that the scurvy is not a disease of the fluids; that its seat is in the muscular fibres; that its proximate cause consists in a gradual diminution of the vital power by the remote causes of this disease; that the torpor, weakness, &c. observed in all the functions are the first effects of the proximate cause, the diminution of the vital power; and that the subsequent diminished cohesion between the particles of the muscular fibres, and the tendency of these to putrefaction, are links of the chain, and are ultimately derived from the same source.

These obfervations on the fcurvy are followed by inquiries into the hiftory of putrid difeafes, which the author thinks will add confirmation to the doctrine juft now advanced.

The feventh chapter contains a concife character of *Typhus* from Dr. Cullen. In the fucceeding chapter, Dr. Milman enumerates the predifpofing caufes of putrid fevers, after which he proceeds to treat of their occafional caufes, and of the means of defeating their operation.

The chief exciting caufe, he fuppofes to be contagion; under which term he includes all forts of matter occafioning putrid fevers, as well that active poifon, which is generated by unclean perfons in confined places, as thofe noxious effluvia which arife from low, marfhy grounds. As the fpecific nature of all fuch matters is entirely unknown to us, and as experience has not yet taught us any antidote by which we can difarm them of their virulence, the prevention of the difeafes, which they are apt to occafion, muft depend principally upon our correction of that ftate of our body by which we are predifpofed to them, and upon our avoiding, as much as poffible, the exciting caufes, at that time efpecially when we feel ourfelves ourselves under the influence of any of the predisposing ones. In the opinion of Dr. Milman, the following advice of Celsus, contains nearly every thing which can be said upon this subject. "Vitare oportet—say he—fatigationem, cruditatem, frigus, calorem, libidinem: tam neque mane surgendum, neque pedibus nudis ambulandum, minimèque post cibum. Cum vero hæc in omni pestilentia facienda sint, tum in eâ maximé quam austri excitarint."

Some of the more modern physicians, however, remarks our author, not content with inculcating the observance of these rules, flatter themselves with the idea of possessing remedies against the exciting causes of putrid fevers. Sylvius pretended to have discovered such a prophylactic in the vegetable acids. Diemerbroeck thought the smoaking tobacco equally efficacious; and the late Sir I. Pringle was of opinion that the circumstance of the plague, pestilential fevers, &c. having abated in Europe during this last century, ought to be ascribed principally to the general use of antiseptics. But that either of these means have a power by any antiseptic or other effect, of disarming the occasional cause of such complaints, or of defending our bodies againſt their impreſ-ſions, Dr. Milman contends is contradicted by the experience of every country. The Turks, he obſerves, ſuffer exceedingly from the plague, though they are in the conſtant uſe of the vegetable acids, and ſmoke more tobacco than other people, and again at Marſeilles, they who purified every thing that was brought to them with vinegar, did not eſcape the plague more than their neighbours who adopted no ſuch precaution.

In treating of the different opinions which have been maintained with reſpect to the nature of the proximate cauſe of fever, which is the ſubject of the tenth chapter, our author quotes the writings of Sydenham, Chenot and others to prove that in the plague and putrid fevers the blood is not in a diſſolved ſtate, and that it is the irritable principle of the body which is chiefly affected by it, whence he concludes that the muſcular fibres are the ſeat of putrid diſeaſes.

In the eleventh chapter our author returns to the ſubject of ſcurvy. It has been matter of much controverſy, whether the ſcurvy be a diſeaſe with which the ancient phyſicians were acquainted, acquainted, or whether it be the production of later times. The affirmative part of this queſtion "that the ſcurvy was both known to and "deſcribed by the ancients," has been maintained by the moſt diſtinguiſhed perſons for their learning from Sennertus to Mead. The authority of Freind ſeems to be the chief ſupport of the negative ſide of the argument; that celebrated writer in his Hiſtory of Phyſic, having, merely on the authority of Fabricius, ſpoken of the ſcurvy as a new diſeaſe, and the offſpring of the 15th century.

Dr. Milman thinks it right to diſcuſs this point, becauſe it may be of conſequence to the doctrine he has advanced, to prove, that, as ſome of the cauſes to which he has referred the ſcurvy are ſuch as may at times prevail in almoſt every country; ſo in fact it has, at ſome period or other, been found to exiſt in moſt parts of the globe. He obſerves that in the Græcian, in the Arabian, and in the Roman authors, we ſhall find many accounts and paſſages, which cannot be deemed to refer to any other complaint. The accounts given by Hippocrates, Paulus Ægineta and Avicenna of the σπλην μεγας are quoted by our author as feeming to afford a prefumption that the difeafe defcribed under that name was no other than the fcurvy. To this he adds a defcription of the Εἰλεὸς αἱματίτης of Hippocrates which he thinks is ftill more agreeable to that of the fcurvy than that even of the σπλην μεγας. The *Stomacace* and the *Sceletyrbe*, difeafes mentioned by Strabo (lib. 16, p. 1127) as having affected the Roman army in an expedition into Arabia under Ælius Gallus, do likewife, in the opinion of our author, correfpond with the known character of the fcurvy, and with the fources from which it is now obferved to arife. The laft quotation he prefents us with on this fubject is from Joinville, an old French writer, who, in his hiftory of St. Louis, is unanimoufly admitted by every one to have given a perfect defcription of the fcurvy which afflicted the Chriftian army in Ægypt in 1260.

In the twelfth and laft chapter, Dr. Milman treats of the cure of the fcurvy and putrid fever. With regard to the fcurvy, the author contents himfelf with enumerating the remedies which have been the moft fuccefsfully employed in this difeafe, by Lind, Kramer, and others. They confift, we are told in fuch things as act upon the fimple folid as nourifhment; and in fuch medicines cines as operate upon the moving fibres, as sudorific, diuretic, stimulant, or tonic remedies. These enquiries naturally lead him to examine Sir John Pringle's theory of antiseptics. The accuracy of the experiments on which that theory was founded, our author does not mean to impeach. It is their application, and the conclusions from them, which he ventures to question. He cannot bring himself to think, that the furnace or the crucible of a chymist affords a fair criterion by which we are to judge of the nature of a medicine; or that the change which it produces on the dead fibres, is to be a rule by which we are to estimate the probable effects of it on the animal machine.

The same principles are applied by our author to the cure of putrid fevers. He observes that substances which have an antiseptic effect on the dead fibres of animals, often produce putrid symptoms in the living body. He sets the experience of Sir John Pringle against his experiments, and appealing to the practice of that learned and candid physician in the jail fever, makes it appear that while his chief views were directed to the supposed putridity of the fluids, his practice was unsuccessful; that when petechiæ

apappeared, and the pulſe began to ſink, Sir John found it neceſſary to vary his method, and to have for his principal intention the ſupport of the vis vitæ; that an accident of a mortification of the back of a man, to whom he had applied a bliſter, having induced him to give the bark at this period, he was led from the advantage derived from it, to give it at the ſame period in other caſes; and that he did this with ſuch aſtoniſhing ſucceſs, that though he was very unfortunate in the event of his former plan of practice, yet that out of thirty-nine caſes he treated in this laſt method, he loſt but four. Reaſoning on theſe facts, our author is led to remark that in caſes of this ſort, the combined powers of camphire, ſerpentaria and contrayerva, though they ſtand foremoſt in the liſt of antiſeptics, are inefficacious; bark, which is inferior to them in antiſeptic virtue, exceeds them in its effect as a remedy. This he accounts for by referring its effect to its action as a tonic on the nervous fibre, while it is yet in the ſtomach, before it can exert any preſervative virtues on their mixture. So quick, he adds, is its operation in fevers, that Sir John Pringle himſelf confeſſes for that reaſon, that that its febrifuge qualities muſt be very different from its antiſeptic ones.

Dr. Milman thinks he may be juſtified in concluding, that the habitual and daily uſe of antiſeptics does not prevent putrid diſeaſes; and that the application of the moſt efficacious ones does not cure them: that the vegetable acids are ſerviceable by gently promoting the proper excretions by the ſkin and by the kidneys; and that the bark operates by its tonic ſtrengthening powers.

